# What is all the noise about in interval timing?

**DOI:** 10.1098/rstb.2012.0459

**Published:** 2014-03-05

**Authors:** Sorinel A. Oprisan, Catalin V. Buhusi

**Affiliations:** 1 Department of Physics and Astronomy, College of Charleston, 66 George Street, Charleston, SC 29624, USA; 2 Department of Psychology, Utah State University, 2810 Old Main Hill, Logan, UT 84332-2810, USA

**Keywords:** striatal beat frequency, interval timing, noise

## Abstract

Cognitive processes such as decision-making, rate calculation and planning require an accurate estimation of durations in the supra-second range—interval timing. In addition to being accurate, interval timing is scale invariant: the time-estimation errors are proportional to the estimated duration. The origin and mechanisms of this fundamental property are unknown. We discuss the computational properties of a circuit consisting of a large number of (input) neural oscillators projecting on a small number of (output) coincidence detector neurons, which allows time to be coded by the pattern of coincidental activation of its inputs. We showed analytically and checked numerically that time-scale invariance emerges from the neural noise. In particular, we found that errors or noise during storing or retrieving information regarding the memorized criterion time produce symmetric, Gaussian-like output whose width increases linearly with the criterion time. In contrast, frequency variability produces an asymmetric, long-tailed Gaussian-like output, that also obeys scale invariant property. In this architecture, time-scale invariance depends neither on the details of the input population, nor on the distribution probability of noise.

## 1. Introduction

**Figure 1.**
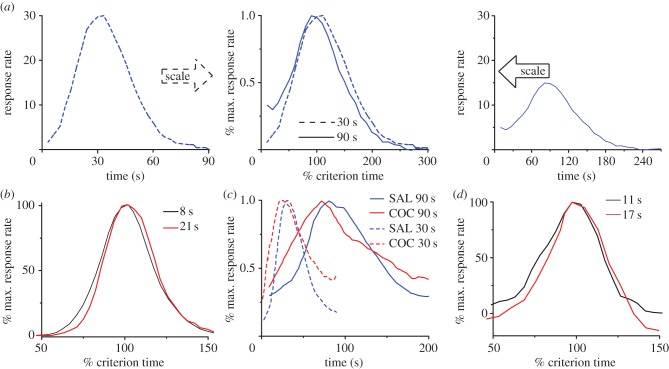
Accurate and time-scale invariant interval timing. (*a*) The response rate of rats timing a 30 s (left) or 90 s interval (right) overlap (centre) when the vertical axis is normalized by the maximum response rate and the horizontal axis by the corresponding criterion time; redrawn from [8]. (*b*) Time-scale invariance in human subjects for 8 and 21 s criteria; redrawn from [9]. (*c*) Systemic cocaine (COC) administration speeds-up timing proportional (scalar) to the original criteria, 30 and 90 s; redrawn from [8]. (*d*) The hemodynamic response associated with a subject's active time reproduction scales with the timed criterion, 11 versus 17 s; redrawn from [10]. An important feature of the output function is its asymmetry, which is clearly visible in (*c*). Although all output functions have a Gaussian-like shape they also present a long tail. (Online version in colour.)

The perception and use of durations in the seconds-to-hours range (interval timing) is essential for survival and adaptation, and is critical for fundamental cognitive processes such as decision-making, rate calculation and planning of action [1]. The classic interval timing paradigm is the fixed-interval (FI) procedure in which a subject's behaviour is reinforced for the first response (e.g. lever press) made after a pre-programmed interval has elapsed since the previous reinforcement. Subjects trained on the FI procedure typically start responding after a fixed proportion of the interval has elapsed despite the absence of any external time cues. A widely used discrete-trial variant of FI procedure is the peak-interval (PI) procedure [2,3]. In the PI procedure, a stimulus such as a tone or light is turned on to signal the beginning of the to-be-timed interval and in a proportion of trials the subject's first response after the criterion time is reinforced. In the remainder of the trials, known as probe trials, no reinforcement is given, and the stimulus remains on for about three times the criterion time. The mean response rate over a very large number of trials has a Gaussian shape whose peak measures the accuracy of criterion time estimation and the spread of the timing function measures its precision. In the vast majority of species, protocols and manipulations to date, interval timing is both *accurate* and *time-scale invariant*, i.e. time-estimation errors increase linearly with the estimated duration [4–7] (figure 1). Accurate and time-scale invariant interval timing was observed in many species [1,4] from invertebrates to fish, birds and mammals such as rats [8] (figure 1*a*), mice [11] and humans [9] (figure 1*b*). Time-scale invariance is stable over behavioural (figure 1*b*), lesion [12], pharmacological [13,14] (figure 1*c*) and neurophysiological manipulations [10] (figure 1*d*).

One of the most influential interval timing paradigms assumes a pacemaker–accumulator clock (pacemaker-counter) and was introduced by Treisman [15]. According to Treisman [15], the interval timing mechanism that links internal clock to external behaviour also requires some kind of store of reference times and some comparison mechanism for time judgement. The model was rediscovered two decades later and became the scalar expectancy theory (SET) [5,16]. SET also assumes that interval timing emerges from the interaction of three abstract blocks: clock, accumulator (working or short-term memory) and comparator. The clock stage is a Poisson process whose pulses are accumulated in the working memory until the occurrence of an important event, such as reinforcement. At the time of the reinforcement, the number of clock pulses accumulated is transferred from the working (short-term) memory and stored in a reference (or long-term) memory. According to the SET, a response is produced by computing the ratio between the value stored in the reference memory and the current accumulator total. To account for the scalar property of interval timing, i.e. the variability of responses is roughly proportional to the peak time, Gibbon [17] showed that a Poisson distribution for the accumulator requires a time-dependent variance in the 'decision and memory factors as well as in the internal clock. These additional sources will be seen to dominate overall variance in performance’ (p. 191), emphasizing the important role of cognitive systems in time judgements. For such reasons, SET was considered more a general theory of animal cognition than strictly a theory of animal timing behaviour [18].

Another influential interval timing model is the behavioural timing (BeT) theory [19,20]. BeT assumes a ‘clock’ consisting of a fixed sequence of states with the transition from one state to the next driven by a Poisson pacemaker. Each state is associated with different classes of behaviour, and the theory claims these behaviours serve as discriminative stimuli that set the occasion for appropriate operant responses (although there is not a one-to-one correspondence between a state and a class of behaviours). The added assumption that pacemaker rate varies directly with reinforcement rate allows the model to handle some experimental results not covered by SET, although it has failed some specific tests (see [21] for a review).

A handful of neurobiologically inspired models explain *accurate* timing and *time-scale invariance* as a property of the information flow in the neural circuits [22,23]. Buonomano & Merzenich [24] implemented a neural network model with randomly connected circuits representing cortical layers 4 and 3 in order to mimic the temporal-discrimination task in the tens to hundreds of milliseconds range. Durstewitz hypothesized that the climbing rate of activity observed experimentally, e.g. from thalamic neurons recordings [25], may be involved in timing tasks [26]. Durstewitz [26] used a single-cell computational model with a calcium-mediated feedback loop that self-organizes into a biophysical configuration which generates climbing activity. Leon & Shadlen [27] suggested that the scalar timing of subsecond intervals may also be addressed at the level of single neurons, though how such a mechanism accounts for timing of supra-second durations is unclear. A solution to this problem was offered by Killeen & Taylor [28] who explained timing in terms of information transfer between noisy counters, although the biological mechanisms were not addressed.

Population clock models of timing are based on the repeatable patterns of activity of a large neural network that allow identification of elapsed time based on a ‘snapshot’ of neural activity [29,30]. In all population clock models, timing is an emergent property of the network in the sense that it relies on the interaction between neurons to produce accurate timing over a time-scale that far exceeds the longest firing period of any individual neuron. The first population clock model was proposed by Mauk and co-workers [7,31,32] in the context of the cerebellum. Such models consist of possible multiple layers of recurrently connected neural networks, i.e. networks of all-to-all coupled neurons that make it possible for a neuron to indirectly feedback onto itself [30]. Depending on the coupling strengths, the recurrent neural networks can self-maintain reproducible dynamic patterns of activity in response to a certain input. Such autonomous and reproducible patterns of neural activity could offer a reliable model for timing. Another advantage of the population clock models is that for weak couplings the network cannot self-maintain reproducible patterns of activity but instead produces input-dependent patterns of activity. Such a model was recently proposed for sensory timing [30]. Similar firing rate models were used by Itskov *et al.* [33] to design a large recurrently connected neural network that produced precise interval timing. By balancing the contribution of the deterministic and stochastic coupling strengths they showed that the first layer of such a population clock model can produce either a reproducible pattern of activity (associated with a timing ‘task’) or desynchronized pattern of activity that cannot keep track of long time-intervals (‘home cage’) [33]. The rate model of Itskov *et al.* [33] was also capable of extracting accurate interval timing information from a second layer with no recurrent excitation and only a global, non-specific recurrent inhibition. The second layer was driven by both the output of the previous layer (through sparse and random connections) and noise [33].

**Figure 2.**
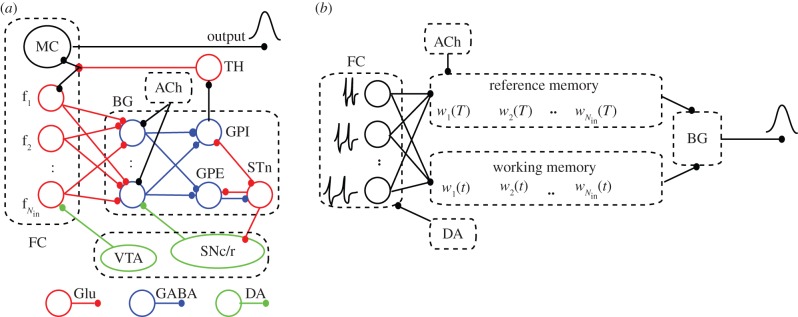
The neurobiological structures involved in interval timing and the corresponding simplified SBF architecture. (*a*) Schematic of some neurobiological structures involved in interval timing. The colour-coded connectivities among different areas emphasize appropriate neuromodulatory pathways. The two main areas involved in interval timing are frontal cortex and basal ganglia. (*b*) In our implementation of the SBF model, the states of the *N*_in_ cortical oscillators (input neurons) at reinforcement time *T* are stored in the reference memory as a set of weights *w_i_*. During test trials, the working memory stores the state of FC oscillators *v_i_*(*t*) and, together with the reference memory, projects its content onto *N*_out_ spiny (output) neurons of the BG. FC, frontal cortex; MC, motor cortex; BG, basal ganglia; TH, thalamus. GPE, globus pallidus external; GPI, globus pallidus internal; STn, subthalamic nucleus; SNc/r, substantia nigra pars compacta/reticulata; VTA, ventral tegmental area; Glu, glutamate; DA, dopamine; GABA, gamma-aminobutyric acid; ACh, acetylcholine. (Online version in colour.)

Finally, a quite different solution was offered by Meck and co-workers [4,34] (figure 2*a*), who proposed the striatal beat frequency (SBF) in which timing is coded by the coincidental activation of neurons, which produces firing beats with periods spanning a much wider range of durations than single neurons [35]. As Matell & Meck [34] suggested, the interval timing could be the product of multiple and complementary mechanisms. They suggested that the same neuroantomical structure could use different mechanisms for interval timing.

Here, we showed analytically that in the context of the proposed SBF neural circuitry, time-scale invariance emerges naturally from variability (noise) in models' parameters. We also showed that time-scale invariance is independent of both the type of the input neuron and the probability distribution or the sources of the noise. We found that the criterion time noise produces a symmetric Gaussian output that obeys scalar property. On the other hand, the frequency noise produces an asymmetric Gaussian-like output with a long tail that also obeys scalar property.

## 2. The striatal beat frequency model

### (a) Neurobiological justification of a striatal beat frequency model

Our paradigm for interval timing is inspired by the SBF model [4,34], which assumes that durations are coded by the coincidental activation of a large number of cortical (input) neurons projecting onto spiny (output) neurons in the striatum that selectively respond to particular reinforced patterns [36–38] (figure 2*a*).

#### (i) Neural oscillators

A key assumption of the SBF model is the existence of a set of neural oscillators able to provide the time base for the interval timing network. There is strong experimental evidence that oscillatory activity is a hallmark of neuronal activity in various brain regions, including the olfactory bulb [39–41], thalamus [42,43], hippocampus [44,45] and neocortex [46]. Cortical oscillators in the alpha band (8–12 Hz [47,48]) were previously considered as pacemakers for temporal accumulation [49], as they reset upon occurrence of the to-be-remembered stimuli [50]. In the SBF model, the neural oscillators are loosely associated with the frontal cortex (FC; figure 2*a*).

#### (ii) Working and long-term memories

Among the potential areas involved in storing brain's states related to salient features of stimuli in interval timing trials are the hippocampus (see [51] and references therein) and the striatum, which we mimic in our simplified neural circuitry (figure 2*a*).

#### (iii) Coincidence detection with spiny neurons

Support for the involvement of the striato-frontal dopaminergic system in timing comes from imaging studies in humans [52–55], lesion studies in humans and rodents [56,57], and drug studies in rodents [58,59] all pointing towards the basal ganglia (BG) as having a central role in interval timing (see also [60] and references therein). Striatal firing patterns are peak-shaped around a trained criterion time, a pattern consistent with substantial striatal involvement in interval timing processes [61]. Lesions of striatum result in deficiencies in both temporal-production and temporal-discrimination procedures [62]. There are also neurophysiological evidences that striatum can engage reinforcement learning to perform pattern comparisons (reviewed by Sutton & Barto [63]). Another reason we ascribed the coincidence detection to medium spiny neurons is due to their bistable property that permits selective filtering of incoming information [64,65]. Each striatal spiny neuron integrates a very large number of afferents (between 10 000 and 30 000) [36,37,65], of which a vast majority (≈ 72%) are cortical [47,66].

#### (iv) Biological noise and network activity

The activity of any biological neural network is inevitably affected by different sources of noise, e.g. channel gating fluctuations [67,68], noisy synaptic transmission [69] and background network activity [70–72]. Single-cell recordings support the hypothesis that irregular firing in cortical interneurons is determined by the intrinsic stochastic properties (channel noise) of individual neurons [73,74]. At the same time, fluctuations in the presynaptic currents that drive cortical spiking neurons have a significant contribution to the large variability of the interspike intervals [75,76]. For example, in spinal neurons, synaptic noise alone fully accounts for output variability [75]. Additional variability affects either the storage (writing) or retrieval (reading) of criterion time to or from the memory [77,78]. Another source of criterion time variability comes from considerations of how animals are trained [79,80]. In this paper, we were not concerned with the biophysical mechanisms that generated irregular firing of cortical oscillators nor did we investigate how reading/writing errors of criterion time happened. We rather investigated whether the above assumed variabilities in the SBF model's parameters can produce *accurate* and *time-scale invariant* interval timing.

### (b) Numerical implementation of a striatal beat frequency model

#### (i) Neural oscillators

Neurons that produce stable membrane potential oscillations are mathematically described as limit cycle oscillators, i.e. they pose a closed and stable phase space trajectory [81]. Because the oscillations repeat identically, it is often convenient to map the high-dimensional space of periodic oscillators using a phase variable that continuously covers the interval (0, 2*π*). Phase oscillator models have a series of advantages: (i) they provide analytical insights into the response of complex networks; (ii) any neural oscillator can be reduced to a phase oscillator near a bifurcation point [82]; and (iii) they allow numerical checks in a reasonable time. All neurons operate near a bifurcation, i.e. a point past which the neuron produces large membrane potential excursions—called action potentials [81].

In this SBF-sin implementation, the cortical neurons, presumably localized in the FC (figure 2*a*), are represented by *N*_in_ (input) phase oscillators with intrinsic frequencies *f_i_*(*i* = 1, *…* , *N*_in_) uniformly distributed over the interval (*f*_min_, *f*_max_), projecting onto *N*_out_ (output) spiny neurons [34] (figure 2*b*). A sine wave is the simplest possible phase oscillator that mimics periodic transitions between hyperpolarized and depolarized states observed in single-cell recordings. For analytical purposes, the membrane potential of the *i*th cortical neuron was approximated by a sine wave *v_i_*(*t*) = *a*cos(2*πf_i_t*), where *a* is the amplitude of oscillations. We also implemented an SBF-ML network in which the input neurons are conductance-based Morris–Lecar (ML) model neurons with two state variables: membrane potential and a slowly varying potassium conductance [83,84] (see electronic supplementary material, section A for detailed model equations).

#### (ii) Working and long-term memories

The memory of the criterion time *T* is numerically modelled by the set of state parameters (or weights) *w_ij_* that characterize the state of cortical oscillator *i* during the FI trial *j*. In our implementation of the noiseless SBF-sin model, the weights 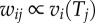, where *T_j_* is the stored value of the criterion time *T* in the FI trial *j*. The state of FC oscillators *i* at the reinforcement time *T_j_* was implemented as the normalized average over all memorized values *T_j_* of the criterion time: 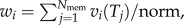 where we used norm = 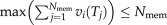 such that the normalized weight is bounded *|w_i_|* ≤ 1 (figure 2*b*). We found no difference between the response of the SBF model with the above weights or the positively defined weight 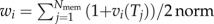.

#### (iii) Coincidence detection with spiny neurons

The comparison between a stored representation of an event, e.g. the set of the states of cortical oscillators at the reinforcement (criterion) time *w_i_*, and the current state *v_i_*(*t*) of the same cortical oscillators during the ongoing test trial is believed to be distributed over many areas of the brain [85]. Based on neurobiological data, in our implementation of the striato-cortical interval timing network, we have a ratio of 1000 : 1 between the input (cortical) oscillators and output (spiny) neurons in the BG (figure 2*b*). The output neurons, which mimic the spiny neurons in the BG, act as coincidence detectors: they fire when the pattern of activity (or the state of cortical oscillators) *w_i_*(*t*) at the current time *t* matches the memorized reference weights *w_i_*. Numerically, the coincidence detection was modelled using the product of the two sets of weights:2.1
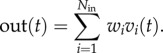


The purpose of the coincidence detection given by equation (2.1) is to implement a rule that produces a strong output when the two vectors *w_i_* and *v_i_*(*t*) coincide and a weaker responses when they are dissimilar. Although there are many choices, such as sigmoidal functions (which involve numerically expensive calculations owing to exponential functions involved), we opted for implementing the simplest possible rule that would fulfil the above requirement, i.e. the dot product of the vectors *w_i_* and *v_i_*(*t*). Without reducing the generality of our approach, and in agreement with experimental findings [66], for analytical analyses, we considered only one output neuron (*N*_out_ = 1) in equation (2.1).

#### (iv) Biological noise and network activity

Two sources of variability (noise) were considered in this SBF implementation. (i) Frequency variability, which was modelled by allowing the intrinsic frequencies *f_i_* to fluctuate according to a specified probability density function pdf*_f_*, e.g. Gaussian, Poisson, etc. Computationally, the noise in the firing frequency of the respective neurons was introduced by varying either the frequency, *f_i_* (in the SBF-sin implementation), or the bias current *I*_bias_ required to bring the ML neuron to the excitability threshold (in the SBF-ML implementation). (ii) Memory variability was modelled by allowing the criterion time *T* to be randomly distributed according to a probability density function pdf*_T_*.

## 3. Results

### (a) No time-scale invariance in a noiseless striatal beat frequency model

In the absence of noise (variability) in the SBF-sin model, the output given by equation (2.1) for *N*_out_ = 1 is (see electronic supplementary material, section B for detailed calculations):3.1
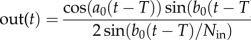
where *a*_0_ = *π* (*f*_max_ + *f*_min_ *−* d*f*), *b*_0_ = *π* (*f*_max_ *− f*_min_), and oscillators' frequencies were equally spaced over the frequency range [*f*_min_, *f*_max_], i.e. *f_k_* = *f*_min_ + *k*d*f* with d*f* = (*f*_max_ *− f*_min_)/*N*_in_ and *k* = 0, *…* , *N*_in_ *−* 1. The envelope of the output function is given by the slowly oscillating function sin(*b*_0_(*t* − *T*))/(2sin(*b*_0_(*t* − *T*)/*N*_in_)).

The width, *σ*_out_, of the output function is determined from the condition that the output function amplitude at *t* = *T* + *σ*_out_/2, i.e. out(*t* = *T* + *σ*_out_/2), is half of its maximum possible amplitude, i.e. 1/2_out_(*t* = *T*). Based on equation (3.1), we predicted theoretically that in the absence of noise *σ*_out_ is independent of the criterion time and violates time-scale invariance (see electronic supplementary material, section B for detailed calculations).

**Figure 3.**
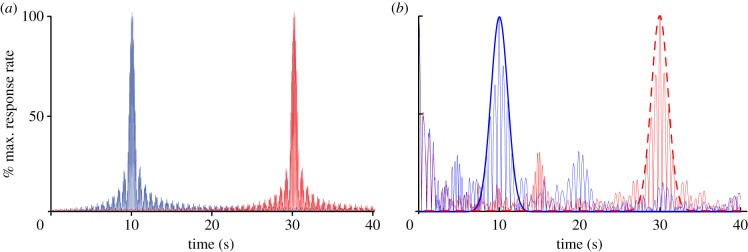
A noise-free SBF model does not produce time-scale invariance. Numerically generated output of a noise-free SBF-sin (*a*) and SBF-ML (*b*) model with *N*_in_ = 1000 for *T* = 10 and *T* = 30 s. As predicted, the width the output function of any noise-free SBF model is independent of the criterion time. The Gaussian envelopes are also shown with continuous line (for *T* = 10 s) and dashed line (for *T* = 30 s). (Online version in colour.)

To numerically verify the above predictions, the envelope of the output function of a noise-free SBF-sin model was fitted with a Gaussian whose mean and standard deviations were contrasted against the theoretically predicted values (figure 3*a*). The width of the envelope is constant regardless of the criterion time and it matches the theoretical prediction.

The above result regarding the emergence of time-scale property from noise in the SBF-sin model can extend to any type of input neuron. Indeed, according to Fourier's theory, any periodic train of action potentials can be decomposed into discrete sine-wave components. It results that irrespective of the type of input neuron, a noise-free SBF model cannot produce time-scale invariant outputs. We verified this prediction by replacing the sine-wave oscillator inputs with biophysically realistic *noise-free* ML neurons (figure 3*b*). Numerical simulations confirmed that the envelope of the output function of the SBF-ML model can be reasonably fitted by a Gaussian (see [48,86,87]), but the width of the Gaussian output does not increase with the timed interval (figure 3*b*), thus violating the time-scale invariance (scalar property).

### (b) Time-scale invariance emerges from criterion time noise in the striatal beat frequency model

Many sources of noise (variability) may affect the functioning of an interval timing network, such as small fluctuations in the intrinsic frequencies of the inputs, and in the encoding and retrieving the weights *w_i_*(*T*) by the output neuron(s) [34,35,86–88]. Here, we showed analytically that one noise source is sufficient to produce time-scale invariance [34,48]. Without compromising generality, in the following, we examined the role of the variability in encoding and retrieval of the criterion time by the output neuron(s). The cumulative effect of all noise sources (trial-to-trial variability, neuromodulatory inputs, etc.) on the memorized weights *w_i_* was modelled by the stochastic variable *T_j_* distributed around *T* according to a given pdf*_T_*. For *N*_out_ = 1, the output function given by equation (2.1) becomes (see electronic supplementary material, section C for detailed calculations):3.2
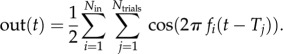
Based on the central limit theorem, the output function given by equation (3.2), which is a sum of a (very) large number *N*_trials_ of stochastic variables *T_j_*, is always a Gaussian, regardless of the pdf*_T_* of the criterion time. We used the estimated value of stochastic output function given by equation (3.2) and found that (see electronic supplementary material, section C for detailed calculations): (i) the output function is always Gaussian (based on the central limit theorem), (ii) peaks at *t*_0*T*_ = *T*(1 + *γ_T_*) ≈ *T* and (iii) the standard deviation of the output function *σ*_out_ is proportional to the criterion time *T*, i.e. obeys the scalar property.

### (c) Particular case: infinite frequency range and time-scale invariance in the presence of Gaussian noise affecting the memorized criterion time

Although we already showed that the output function for the SBF-sin model and arbitrary pdf*_T_* for the criterion time noise is always Gaussian, produces accurate interval timing and obeys scalar property, it is illuminating to grasp the meaning of the theoretical coefficients in our general result by investigating a biologically relevant particular case. If the criterion time is affected by a Gaussian noise with zero mean and standard deviation *σ_T_*, then one can show that (see electronic supplementary material, section D for detailed calculations), in the limit of a very large pool (theoretically infinite) of inputs, the output function of the SBF-sin model is3.3
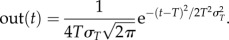


The output function given by equation (3.3) with the physically realizable term centred at *t* = *T*: (i) has a Gaussian (as predicted by the central limit theorem), (ii) peaks at *t* = *T*, i.e. produces accurate timing and (iii) has a standard deviation3.4
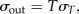
which obeys scalar property. We previously showed that for arbitrary noise distributions affecting the criterion time, the peak of the output function should be at *t*_0*T*_ = *T*(1 + *γ_T_*). The actual peak in the presence of the Gaussian noise is at *t* = *T*, which shows that in this particular case *γ_T_* = 0.

### (d) Particular case: finite frequency range and time-scale invariance in the presence of Gaussian noise affecting the memorized criterion time

In our previous numerical implementations of the SBF model [48,86,87], the frequency range was finite and coincides with *α* band (8–12 Hz). Is the SBF model still performing accurate and scalar interval timing under such a strong restriction? For a finite range of frequencies (*f*_min_ < *f* < *f*_max_) with a very large number of FC oscillators *N*_in_, a more realistic estimation of the output function from equation (3.2) is (see electronic supplementary material, section E for detailed calculations):3.5
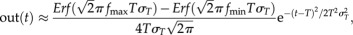
where *Erf*() is the error function. A major difference between the equation (3.3), which is valid in the limit of an infinite range of frequencies of the FC oscillators, and the equation (3.5), which takes into account the fact that there is always only a finite frequency range of FC oscillators, is the frequency-dependent amplitude of the output function represented by the difference of the two *Erf*() functions. Therefore, we proved that even for a finite frequency range of the FC oscillators the output function given by equation (3.5) is (i) Gaussian (ii) centred on the criterion time *T*, and obeys scalar property with a width3.6
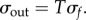


**Figure 4.**
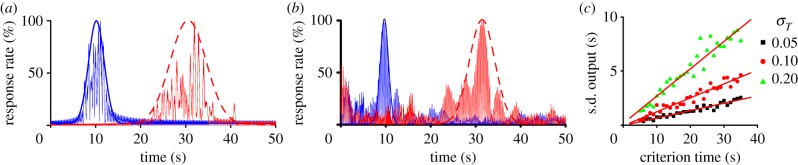
Time-scale invariance emerges from criterion time noise in the SBF model. (*a*) Time-scale invariance emerges spontaneously in a noisy SBF-sin model; here, the two criteria are *T* = 10 and *T* = 30 s. The output functions (thin continuous lines) were fitted with Gaussian curves (thick continuous line for *T* = 10 s and dashed line for *T* = 30 s) in order to estimate the position of the peak and the width of the output function. In an SBF-sin model, the standard deviation of the output function increases linearly with the criterion time. (*b*) In an SBF-ML implementation, the output function still has a Gaussian shape (owing to central limit theorem) and its width increases with criterion time. (*c*) Numerical simulations confirm that the standard deviation of the output function *σ*_out_ increases linearly with the criterion time *T*, which is the hallmark of time-scale invariance. Furthermore, for Gaussian memory variance we also found that *σ*_out_ is proportional to the standard deviation of the noise *σ_T_*. (Online version in colour.)

We used the SBF-sin implementation to numerically verify our theoretical prediction that *σ*_out_ = *Tσ_f_* over multiple trials (runs) of this type of stochastic process and for different values of *T*. The output functions (see continuous lines in figure 4*a*) for *T* = 10 s and *T* = 30 s are reasonably fitted by Gaussian curves. Our numerical results show a linear relationship between *σ*_out_ of the Gaussian fit of the output and *T*. We found that the resultant slope of this linear relationship matched the theoretical prediction given by *σ*_out_ = *Tσ_f_*. For example, for *σ_T_* = 10% the average slope was 11.3% ± 4.5% with a coefficient of determination of *R*^2^ = 0.93, *p* < 10*^−^*^4^. We also found that for the SBF-ML the width of the Gaussian envelope increases linearly with the criterion time (figure 4*b*). For example, figure 4*c* shows the slope of the standard deviation *σ*_out_ versus criterion time for different values of the standard deviation of the Gaussian noise. Figure 4*c* shows not only that the scalar property is valid, but it also shows that 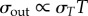 as we predicted theoretically. Indeed, for *σ_T_* = 0.05 the numerically estimated proportionality constant is 0.068 (filled squares in figure 4*c*, *R*^2^ = 0.97) for *σ_T_* = 0.1 the slope is 0.129 (filled circles in figure 4*c*, *R*^2^ = 0.96) and for *σ_T_* = 0.2 the slope is 0.25 (filled triangles in figure 4*c*, *R*^2^ = 0.96).

### (e) Time-scale invariance emerges from frequency variance during probe trials in the striatal beat frequency model

In addition to memory variance, frequency fluctuations owing to stochastic channel noise or background networks activity has received considerable attention. Here, we considered only frequency variability during the probe trial and assumed that there was no frequency variability during the FI procedure while the weights *w_i_* were memorized. We also assumed that there is no variability in the memorized criterion time, because its effect on interval timing was already addressed in §3*d*.

The cumulative effect of all noise sources on the firing frequencies during the probe trials was modelled by the stochastic variable *f_ij_* distributed around the frequency *f_i_* according to a given pdf*_f_*. Based on equation (2.1) with *N*_out_ = 1, the output function term centred around *t* = *T* becomes (see electronic supplementary material, section F for detailed calculations):3.7
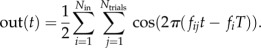


Based on the central limit theorem, the output function given by equation (3.7), which is the sum of a (very) large number *N*_trials_ of stochastic variables *f_ij_*, is always a Gaussian, regardless of the pdf*_f_*. We used the average value of the stochastic equation (3.7) to estimate the output function and found that (see electronic supplementary material, section F for detailed calculations) it is always: (i) Gaussian (based on the central limit theorem), (ii) peaks at *t*_0*f*_ = *T*/(1 + γ*_f_*) ≈ *T* and (iii) has a standard deviation *σ*_out_ that increases linearly with the criterion time *T*3.8
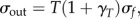
i.e. obeys scalar property.

### (f) Particular case: infinite frequency range and time-scale invariance in the presence of Gaussian noise affecting oscillators’ frequencies during probe trials

**Figure 5.**
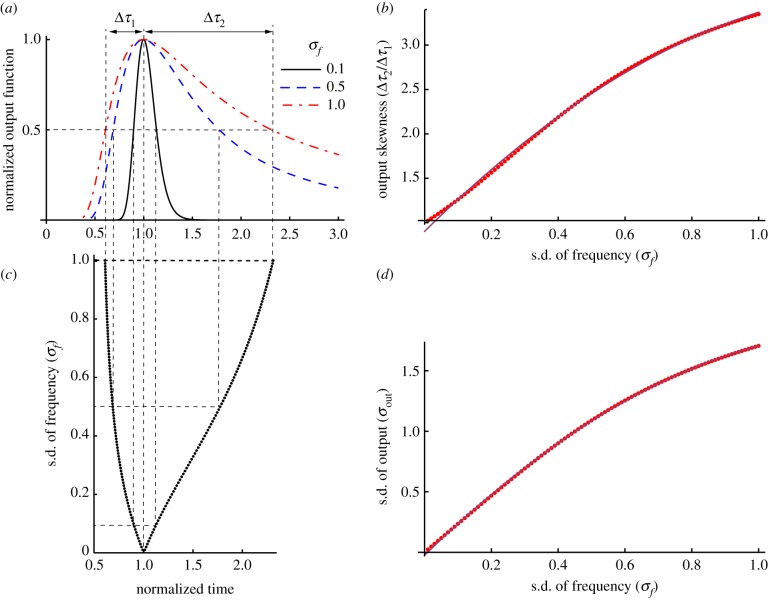
Frequency noise produces a skewed Gaussian-like output with a long tail—theoretical predictions. (*a*) Theoretically predicted output function for Gaussian noise affecting the oscillators' frequencies is a skewed Gaussian-like output. The normalized output function is plotted against the normalized time *t*/*t*_0*f*_, where *t*_0*f*_ is the time marker for the peak of the output function. The skewness is measured by the two corresponding half-widths *τ*_1_ and *τ*_2_. (*b*) Although both half-widths increase with the standard deviation of the frequency noise, the long tail of the output is determined by the very fast increase of *τ*_2_. (*c*) A quantitative measure of the skewness is the ratio *τ*_2_/*τ*_1_, which increases faster than linearly with *σ_f_*. (*d*) The width of the output function *σ*_out_ also increases faster than linearly with *σ_f_*. (Online version in colour.)

As in §2*e*, we used a Gaussian distribution pdf*_f_* to explicitly compute the theoretical coefficients in the above general result. Briefly, by replacing the stochastic frequencies *f_ij_* with an appropriate Gaussian distribution *f_i_*(1 + Gauss(0, *σ_f_*)*_j_*), we found that the output function is (see electronic supplementary material, section G for detailed calculations):3.9
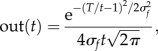
which looks like a Gaussian with a very long tail (figure 5*a*) and peaks at 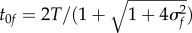. The skewness of the output function increases with the standard deviation of the frequency noise *σ_f_*. For *t* < *t*_0*f*_, the half-width Δ*τ*_1_ increases with the standard deviation of the frequency noise *σ_f_*, although at a much slower rate than Δ*τ*_2_ for *t* > *t*_0*f*_ (figure 5*b*). This fact is reflected in a faster than linear increase of the Δ*τ*_2_/Δ*τ*_1_ against *σ_f_* (figure 5*c*). The quadratic fit over the entire *σ_f_ ∈* [0, 1] shown in figure 5*c* is given by Δ*τ*_2_/Δ*τ*_1_ = (0.902 ± 0.007) + (3.74 ± 0.03)*σ_f_* + (−1.27 ± 0.03)*σ_f_*^2^ with an adjusted *R*^2^ = 0.999. For reasonable standard deviation of the frequency noise *σ_f_* < 0.5, we found that 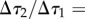 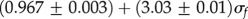 with an adjusted *R*^2^ = 0.999. As the output function given by equation (3.9) is no longer symmetric with respect to the peak located at 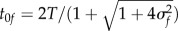, the width of the output function is given 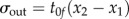, where *x*_1_ and *x*_2_ are the solutions of the half-width equation out(*x*) = 1/2out(1). We found (figure 5*d*) that the width of the output function *σ*_out_ 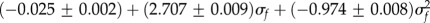 with an adjusted *R*^2^ = 0.9999 over the entire range *σ_f_* ∈ [0, 1]. A reasonable approximation for standard deviation of the frequency noise *σ_f_* < 0.5 is linear *σ*_out_ = (0.019 ± 0.003) + (2.20 ± 0.01)*σ_f_* with an adjusted *R*^2^ = 0.999. As a result, in the presence of frequency variability during probe trials, we predict theoretically that the SBF model (i) produces a Gaussian-like output function with a long tail, (ii) produces accurate interval timing (the output function is centred on 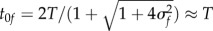) and (iii) obeys scalar property with 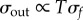. We also noted that the peak time predicted for an arbitrary pdf*_f_*, i.e. *t*_0*f*_ = *T*/(1 + *γ_f_*) is identical with the peak time in the particular case of Gaussian noise, i.e. 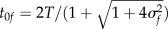 if 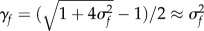 for 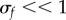.

### (g) Particular case: finite frequency range and time-scale invariance in the presence of Gaussian noise affecting oscillators’ frequencies during probe trials

For a finite range of frequencies (*f*_min_ < *f* < *f*_max_) with a very large number of FC oscillators *N*_in_, a more realistic estimation of the output function from equation (3.7) is (see electronic supplementary material, section H for detailed calculations):3.10
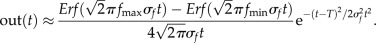


A significant difference between equation (3.9), which is valid in the limit of an infinite frequency range of the FC oscillators, and equation (3.10), which takes into consideration that there is always only a finite frequency range of the FC oscillators, is the frequency-dependent factor in the output function represented by the difference of the two *Erf*() functions. The output function in equation (3.10) resembles a Gaussian with a long tail and obeys time-scalar invariance property.

**Figure 6.**
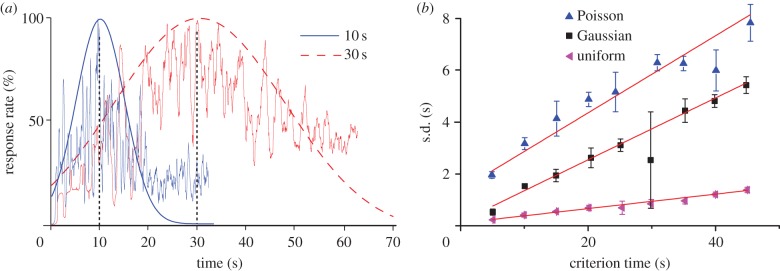
Frequency noise produces a skewed Gaussian-like output with a long tail in numerical results. (*a*) The output function (thin continuous lines) of the SBF-ML model for *T* = 10 and *T* = 30 s has a Gaussian shape and its peak can be reasonable localized by a Gaussian fit (thick continuous line for *T* = 10 and dashed line for *T* = 30 s). The effect of frequency noise is the asymmetric output function that has a long tail. (*b*) The width of the output function increases linearly with the criterion time and obey the time-scale invariance property. (Online version in colour.)

We used both the SBF-sin and SBF-ML implementations to numerically verify that (i) the output function resembles a Gaussian with a long tail (figure 6*a*), and (ii) the width of the output function increases linearly with the criterion time (figure 6*b*). The output functions of the SBF-ML implementation (see thin continuous lines in figure 6*a*) for *T* = 10 s and *T* = 30 s are reasonably fitted by Gaussian curves (see thick continuous line for *T* = 10 s and dashed line for *T* = 30 s in figure 6*a*). However, as predicted theoretically, the output has a long tail. The scalar property is indeed valid, because the width of the output function linearly increase with the criterion time (figure 6*b*).

Furthermore, we checked that the scalar property holds not only for Gaussian noise, which allowed us to determine an analytic expression for the long-tailed output function in §4*f*, but also for uniform and Poisson noise.

## 4. Discussion

Computational models of interval timing vary largely with respect to the hypothesized mechanisms and the assumptions by which temporal processing is explained, and by which time-scale invariance or drug effects are explained. The putative mechanisms of timing rely on pacemaker/accumulator processes [5,6,89,90], sequences of behaviours [20], pure sine oscillators [8,34,91,92], memory traces [21,93–97] or cell and network-level models [27,98]. For example, both neurometric functions from single neurons and ensembles of neurons successfully paralleled the psychometric functions for the to-be-timed intervals shorter than 1 s [27]. Reutimann *et al.* [99] also considered interacting populations that are subject to neuronal adaptation and synaptic plasticity based on the general principle of firing rate modulation in a single cell. Balancing long-term potentiation (LTP) and long-term depression (LTD) mechanisms are thought to modulate the firing rate of neural populations with the net effect that the adaptation leads to a linear decay of the firing rate over time. Therefore, the linear relationship between time and the number of clock ticks of the pacemaker–accumulator model in SET [5] was translated into a linearly decaying firing rate model that maps time and variable firing rate.

By and large, to address time-scale invariance, current behavioural theories assume convenient computations, rules or coding schemes. Scalar timing is explained as either deriving from computation of ratios of durations [5,6,100], adaptation of the speed at which perceived time flows [20] or from processes and distributions that conveniently scale up in time [21,91,93,95,96]. Some neurobiological models share computational assumptions with behavioural models and continue to address time-scale invariance by specific computations or embedded linear relationships [101]. Some assume that timing involves neural integrators capable of linearly ramping up their firing rate in time [98], whereas others assume LTP/LTD processes whose balance leads to a linear decay of the firing rate in time [99]. It is unclear whether such models can account for time-scale invariance in a large range of behavioural or neurophysiological manipulations.

Neurons are often viewed as communications channels that respond even to the precisely delivered stimuli sequence in a random manner consistent with Gaussian noise [102]. Biological noise was shown to play important functional roles, e.g. enhance signal detection through stochastic resonance [103,104] and stabilize synchrony [105,106]. Firing rate variability in neural oscillators also results from ongoing cortical activity (see [106,107] and references therein), which may appears noisy simply because it is not synchronized with obvious stimuli.

A possible common ground for all interval timing models could be the threshold accommodation phenomenon that allows stimulus selectivity [108,109] and promotes coincidence detection [11]. Farries [110] showed that dynamic threshold change in subthalamic nucleus (STn) that projects to the output nuclei of the BG allows STn to act either as an integrator for rate code inputs or a coincidence detector [110] (figure 2). Interestingly, under both conditions, faulty (noisy) processing explains time-scale invariance. For example, Killeen & Taylor [28] explained scale invariance of counting in terms of noisy information transfer between counters. Similarly, here, we explained time-scale invariance of timing in terms of noisy coincidence detection during timing. Therefore, it seems that when BG acts either as a counter or as coincidence detector, neural noise alone can explain time-scale invariance.

Our theoretical predictions based on an SBF model show that time-scale invariance emerges as the property of a (very) large and noisy network. Furthermore, we showed that the output function of an SBF mode always resembles the Gaussian shape found in behavioural experiments, regardless of the type of noise affecting the timing network. We showed analytically that in the presence of arbitrary criterion variability alone the SBF model produces an output that (i) has a symmetric and Gaussian shape, (ii) is accurate, i.e. the peak of the output is located at *t*_0*T*_ = *T*(1 + γ*_T_*), where 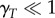 is a constant that depends on the type of memory noise and (iii) has a width that increases linearly with the criterion time, i.e. obeys time-scale invariance property. The memory variability is ascribed to storing or retrieving the representation of criterion time to and from the long-term memory (figure 2*b*). We also showed analytically and verified numerically that for a Gaussian noise affecting the memory of the criterion time the output function of SBF-sin model is analytic and its peak is at *t*_0*T*_ = *T*, which means that for Gaussian noise γ*_t_* = 0 (figure 4*a*). All of the above properties were also verified by replacing phase oscillators with biophysically realistic ML model neurons (figure 4*b,c*).

We also showed analytically that, in the presence of arbitrary frequency variability alone, the SBF model produces an output that (i) has a Gaussian-like shape (based on the central limit theorem, (ii) is accurate, i.e. the peak of the output is located at *t*_0*f*_ = *T*/(1 + *γ_f_*), where 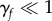 is a constant that depends on the type of frequency noise and (iii) has a width *σ*_out_ = *T*(1+γ_*T*_)*σ*_*f*_ that increases linearly with the criterion time, i.e. obeys time-scale invariance property. In the presence of Gaussian noise, the output function is analytic, asymmetric and Gaussian-like (figure 5*a*) with a skewness that increases quadratically with the standard deviation of the frequency noise (figure 5*b*). In addition to the fact that the standard deviation of the output function is proportional to the criterion time and, therefore, obeys the time-scale invariance property, it also increases quadratically with the standard deviation of the frequency noise (figure 5*d*). For Gaussian noise, the peak of the asymmetric, long-tailed Gaussian-like output (figure 5*a*) resembles experimental data that show a strong long tail in subjects' responses (figure 1*c*).

Our results regarding the effect of noise on interval timing support and extend the speculation [34] by which an SBF model requires at least one source of variance (noise) to address time-scale invariance. Rather than being a signature of higher-order cognitive processes or specific neural computations related to timing, time-scale invariance naturally emerges in a massively connected brain from the intrinsic noise of neurons and circuits [4,27]. This provides the simplest explanation for the ubiquity of scale invariance of interval timing in a large range of behavioural, lesion and pharmacological manipulations.
